# Relaxed Lockdown in Bangladesh During COVID-19: Should Economy Outweigh Health?

**DOI:** 10.34172/ijhpm.2020.98

**Published:** 2020-06-10

**Authors:** Raaj Kishore Biswas, Samin Huq, Awan Afiaz

**Affiliations:** ^1^Transport and Road Safety (TARS) Research Centre, School of Aviation, University of New South Wales, Sydney, NSW, Australia.; ^2^Child Health Research Foundation, Dhaka, Bangladesh.; ^3^Institute of Statistical Research and Training, University of Dhaka, Dhaka, Bangladesh.

## Dear Editor,

The ongoing coronavirus disease 2019/severe acute respiratory syndrome coronavirus 2 (COVID-19/SARS-CoV-2) pandemic has challenged scientists and policy-makers worldwide. As of June 5, 2020, it has spread across 216 countries, infected 6.5 million people and caused 387 155 deaths, and has pushed the boundaries of healthcare infrastructure, forced lockdowns and halted economies. Bangladesh, a country of the global south is no exception, with a total of 60 391 reported cases with 811 deaths since its first diagnosis on 8 March. These estimates have been made based on the 372 365 tests in 52 testing centers across the country as of June 5, 2020.^1^ Official estimates showed that more than 54% of the cases belonging to the age group of 21-40 years and 71% of the cases affecting males but majority of the deaths (68%) are placed in the patients above 50 years old. The recovery rate has been low, only 21.4% of the cases despite a sudden change in the definition of recovery, compared to the 52% of the COVID-19 cases worldwide.^2^

**Figure 1:**
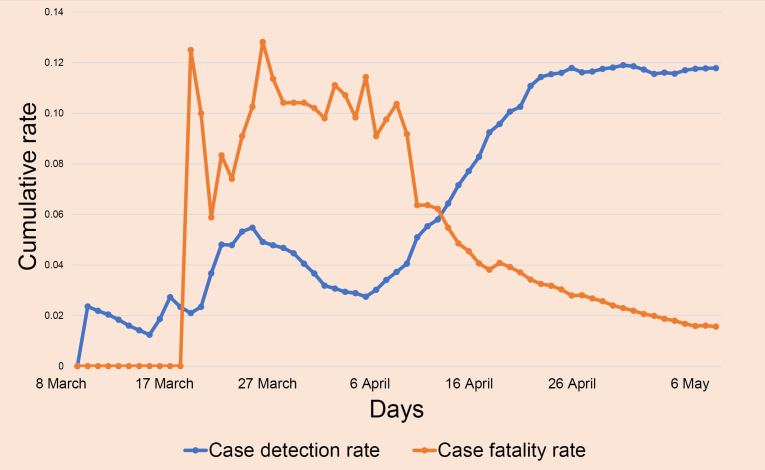
Distribution of Cumulative Reported Cases and Fatalities Based on Total Tests Conducted in Bangladesh From 8 March to 8 May.

**Figure 2:**
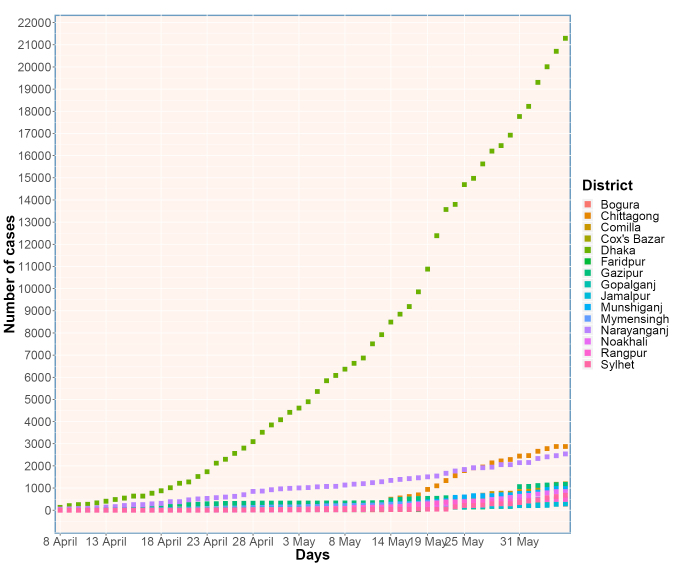
Distribution of Reported Cases in Six Districts That Had Highest Frequency of Cases From 8 April to 8 May.

The exponential growth in infection numbers was observed as the first 1000 cases were detected in 38 days whereas the last 2000 cases were detected in merely 8 days (Figure 1). The rate of spread in Bangladesh has outpaced its neighbors in Southeast Asia. Despite the expansion to 52 testing centers, the testing coverage has remained lower (2338 per million) than neighboring countries such as India, Pakistan or India.^1^ This lack of data clouded the true infection rate, particularly with the majority of testing centers based in Dhaka, the capital, could have contributed to the observed high infection rate in Dhaka (2040/million) and its neighboring cities (Narayanganj 6162/million, Munshiganj 4324/million and Gazipur 15 171 687/million) leading to a delay in testing in rural communities (Figure 2).

Despite the preparation of various guiding documents,^3^ early initiatives such as designated quarantine from high-risk country arrival was hampered due to angry crowd reaction while subsequent home quarantine was derelict due to non-adherence and a lack of monitoring.^4^ Inequitable distribution of limited workforce and supplies coupled with the supply of faulty logistics have compromised the overall health infrastructure including the COVID-19 response, which created doubts in people regarding the system despite iterative recruitment of healthcare workers and establishing of new isolation facilities. The desire for a quick fix contributed to the approval of manufacturing of drugs without sufficient evidence along with limited information on their adverse effects. This puts the population at a higher risk of developing other adverse conditions impacting quality of life post recovery from COVID-19 alongside potential mortality from other diseases.

The National Preparedness and Response Plan for COVID-19 is a good-on-paper strategy that has been poorly implemented till date.^3^ The state response system failed to incorporate community feedback rendering several ineffective government initiatives primarily due to lack of coordination among the regulatory bodies and inability to instigate evidence-based decision-making caused by limited testing capabilities.^5,6^ Harboring misconceptions regarding treatment, stigmatization of COVID-19 patients and irresponsible statements from authorities attributed to a poor communication strategy implementation coupled with the state’s focus on alleviating its criticism on mainstream and social media.^7^ All these amplified by general people’s reluctance to adhere to lockdown measures despite heavy presence of police and armies in the streets.

Early needs assessment suggested only 32% of the healthcare workers were willing to provide services which rendered 45% of the healthcare facilities inaccessible.^8^ This pandemic has severely affected the incomes of 35% of the population with typical monthly income under US$120 without any savings,^8^ which escalated further when 370 ready-made garments (RMGs) factories reportedly did not pay workers their due salaries prior to the shutdown. Moreover, there is a continued threat of factory shutdown and worker layoffs despite the US$8 billion stimulus package from the government. Even though the investment in health was made the highest priority by the Key Informant Assessment, shortage of food security and inadequate sanitation facilities have overshadowed the impact of investment in healthcare at the community level.^8^ Therefore, a section of the population advocated to uplift the lockdown due to their perceived unavailability of income opportunities, especially with the largest religious festival in the last week of May facilitating mass migration across cities which resulted in a spike in the number of reported cases. The government conditionally reopened the service sector based on the ground of maintaining social distancing and offered an option to open shopping malls with the same conditions. Over half of the reported positive cases from RMG workers occurred after the re-opening.^9^

Relaxing the lockdown was an attempt to resuscitate the ailing economy suffering from the pandemic, sparking a national debate. Those in favor warn of a devastated economy, loss of jobs and livelihood and in an extreme case, the possibility of a famine. In contrast, the opposition arguments include a higher spread in infection and subsequent overwhelm in the health system, risking the lives of emergency workers and consequently enforcing the lockdown again and shutting down the economy.

The choice of health versus economy is a preference between quality of life and unknown quantity of collateral damage. The ongoing pandemic and subsequent lockdown is causing nearly US$400 million per day worth of losses in service and agricultural sectors.^10^ However, as of May 8, 2020, 1429 police officers, 97 factory workers and 1168 health professionals tested positive for COVID-19 which accumulated to 20.5% of all reported cases during the lockdown period. An estimated 83% people are suffering from mental distress, which can be attributed to both lockdown and pandemic panic.^8^

The forced re-opening of RMG factories by easing lockdown questions the effectiveness of the stimulus package from the government to address economic slowdown. Interestingly, allowing shopping malls and factories to function without easing the restriction on the public transport system shows the lack of a well-planned strategy. These could be further escalated as the National Task Force for COVID-19 only consists of medical doctors aimed at possible integration of clinical side of the pandemic, where a multi-disciplinary team including health economists, informaticians, public health experts might help envision such policy errors integrating socioeconomic impact and evidence-driven decision-making. The transition to normalcy would require a prepared health system that could treat a high number of infections once the lockdown is relaxed.

To ensure that easing the lockdown does not aggravate the damages, this relaxation strategy could be experimented at the districts with lower case burdens, and so rural agriculture could take precedence over urban industries. This transition strategy could follow successful nations such as New Zealand with their four-level risk assessment system or Australia with the three-stage transition plan to normalcy. Regardless of the strategy Bangladesh takes to restore normalcy, without adequately equipping the health system and a long-term plan of boosting general health literacy rate, economic survival might not be feasible.

## Acknowledgments

The authors would like to acknowledge Institute of Epidemiology, Disease Control and Research (IEDCR), who regularly published COVID-19 data on Bangladesh. We convey thanks to the media for the situational analysis.

## Ethical issues

Publicly available data from Bangladesh Government were used in this study. They would be made available upon requesting the authors.

## Competing interests

Authors declare that they have no competing interests.

## Authors’ contributions

RKB conceptualised the study, synthesised the analysis plan, coded the figures and compiled the manuscript. SH drafted the manuscript and conducted literature review. AA drafted the manuscript, compiled the data, and conducted literature review. The final manuscript was read and approved by all the authors.

## Authors’ affiliations

^1^Transport and Road Safety (TARS) Research Centre, School of Aviation, University of New South Wales, Sydney, NSW, Australia. ^2^Child Health Research Foundation, Dhaka, Bangladesh. ^3^Institute of Statistical Research and Training, University of Dhaka, Dhaka, Bangladesh.

## Funding

This research received no specific grant from any funding agency in the public, commercial, or not-for-profit sectors.

## References

[1] IEDCR. Institute of Epidemiology Disease Control and Research IEDCR COVID-19 Dashboard. https://www.iedcr.gov.bd. 2020.

[2] COVID-19: Govt brings changes in recovery criteria. Prothom Alo website. https://en.prothomalo.com/bangladesh/covid-19-changes-in-recovery-criteria. Published May 9, 2020.

[3] Azad AK. National Preparedness and Response Plan for COVID-19, Bangladesh. Dhaka: Ministry of Health and Family Welfare; 2020.

[4] Fear grows as two test COVID-19 positive in Gaibandha’s Sadullapur. Prothom Alo website. https://en.prothomalo.com/bangladesh/fear-grows-as-two-test-covid-19-positive-in-gaibandhas-sadullapur. March 23, 2020.

[5] Huq S, Biswas RK (2020). COVID-19 in Bangladesh: data deficiency to delayed decision. J Glob Health.

[6] Islam M, Siddika A. COVID-19 and Bangladesh: a study of the public perception on the measures taken by the government. 2020. doi:10.13140/rg.2.2.30042.49608.

[7] Refrain from Negative Remarks Against Important Persons on Social Media: Ministry. The Daily Star. May 7, 2020. https://www.thedailystar.net/refrain-negative-remarks-against-important-persons-social-media-1900411.

[8] Needs Assessment Working Group. COVID-19: Bangladesh Multi-Sectoral Anticipatory Impact and Needs Analysis. https://www.humanitarianresponse.info/en/operations/bangladesh/document/covid-19-bangladesh-multi-sectoral-anticipatory-impact-and-needs. Published April 27, 2020.

[9] The Daily Star. Infected RMG Workers: ‘Half Tested Positive after Factories Reopened’. The Daily Star. May 8, 2020. https://www.thedailystar.net/backpage/news/infected-rmg-workers-half-tested-positive-after-factories-reopened-1900540.

[10] The Financial Express. BD economy loses Tk 33b every day during shutdown: Study. The Financial Express. April 21, 2020. https://thefinancialexpress.com.bd/economy/bangladesh/bd-economy-loses-tk-33b-every-day-during-shutdown-study-1587472624.

